# Selective lysis of breast carcinomas by simultaneous stimulation of sodium channels and blockade of sodium pumps

**DOI:** 10.18632/oncotarget.24581

**Published:** 2018-02-26

**Authors:** Harry J. Gould, Jack Norleans, T. David Ward, Chasiti Reid, Dennis Paul

**Affiliations:** ^1^ Department of Neurology, Louisiana State University Health Sciences Center, New Orleans, LA, USA; ^2^ Department of Pharmacology and Experimental Therapeutics, Louisiana State University Health Sciences Center, New Orleans, LA, USA; ^3^ Department of Anesthesiology, Louisiana State University Health Sciences Center, New Orleans, LA, USA; ^4^ Department of Cell Biology and Anatomy, Louisiana State University Health Sciences Center, New Orleans, LA, USA; ^5^ Stanley S. Scott Cancer Center, Louisiana State University Health Sciences Center, New Orleans, LA, USA; ^6^ Neuroscience Center of Excellence, Louisiana State University Health Sciences Center, New Orleans, LA, USA; ^7^ Center of Excellence for Oral and Craniofacial Biology, Louisiana State University Health Sciences Center, New Orleans, LA, USA

**Keywords:** targeted osmotic lysis, epithelial carcinoma, cancer, sodium channels, sodium pumps

## Abstract

Sodium influx through voltage-gated sodium channels (VGSCs) coupled with balanced removal of sodium ions via Na^+^, K^+^-ATPase is a major determinant of cellular homeostasis and intracellular ionic concentration. Interestingly, many metastatic carcinomas express high levels of these channels. We hypothesized that if excess VGSCs are activated and Na^+^, K^+^-ATPase is simultaneously blocked, the intracellular Na^+^ concentration should increase, resulting in water movement into the cell, causing swelling and lytic cell death. MDA-MB-231 breast cancer cells over-express VGSCs by 7-fold. To test our hypothesis, we treated these cells *in vitro* with the Na^+^, K^+^-ATPase blocker, ouabain, and then stimulated with a sublethal electric current. For *in vivo* histologic and survival studies, MDA-MB-231 xenografts were established in Nu/J mice. Mice injected with saline or ouabain were electrically stimulated with trains of 10 msec 10V DC pulses. Within seconds to minutes, the cells swelled and lysed. MCF-10a cells, which express normal VGSCs levels, were unaffected by this treatment. Cells from the weakly-malignant cell line, MCF-7, which express 3-fold greater VGSCs than MCF-10a cells, displayed an intermediate time-to-lysis. The rate of lysis correlated directly with the degree of sodium channel expression and malignancy. We also demonstrated efficacy in cell lines from prostate, colon and lung carcinomas. Treated MDA-MB-231 xenografts showed 60–80% cell death. In survival studies, TOL-treated mice showed significantly slower tumor growth vs. controls. These results are evidence that this ”targeted osmotic lysis” represents a novel method for selectively killing cancer cells and warrants further investigation as a potential treatment for advanced and end-stage breast cancer.

## INTRODUCTION

Malignant neoplasms remain second only to heart disease as the leading cause of death in the United States [1]. Although early detection and advances in therapeutic technology have significantly improved the 5-year survival rate for cancers diagnosed in early stages, there has been little effect on survival when the disease is diagnosed in advanced and end-stages [2, 3]. Unfortunately, traditional methods for treating malignancy are fraught with serious morbidity and reduction in quality of life due to the toxic effects imposed on both normal and abnormal tissues. We propose a novel and promising approach for treating advanced and end-stage breast cancer that is based on coupling the observation that many of the most aggressive epithelially-derived carcinomas over-express voltage-gated sodium channels (VGSCs) [4–7], and the results of studies we have conducted on the role of VGSCs in the development and maintenance of chronic pain [8–11].

Previously, we observed that after the s.c. injection of complete Freund’s adjuvant (CFA), there is a rapid and dramatic increase in the expression of VGSC protein in the neurons associated with the area of robust and persistent inflammation that is produced [8]. We also observed a commensurate increase in sodium, potassium-ATPase (Na^+^, K^+^-ATPase or ‘sodium pump’) [11]. We have seen VGSC overproduction in excess of 50-fold, whereas the overproduction of sodium pumps has been 2- to 5-fold. Simultaneous blockade of Na^+^, K^+^-ATPase and stimulation of VGSCs produced swelling and lysis of dorsal root ganglion cells that over-expressed VGSCs, but not of the surrounding neurons [11].

Breast cancer is one of the carcinomas that expresses or over-expresses VGSCs [5, 6]. VGSC expression is directly related to a cancer’s aggressiveness and ability to metastasize [12–17]. Accordingly, we reasoned that simultaneous inhibition of Na^+^, K^+^-ATPase function and stimulation of the VGSCs in highly-malignant breast cancer cells would cause excessive influx of sodium ions and commensurately large volumes of water. The increase in cell volume would cause an increase in cellular osmotic pressure and the selective lysis of the neoplastic cells that over-express VGSCs, whereas noncancerous cells that express normal levels of VGSCs would not lyse. We have called this process “targeted osmotic lysis (TOL)”.

## RESULTS

### Relationship between VGSC expression and degree of malignancy

**Figure 1:**
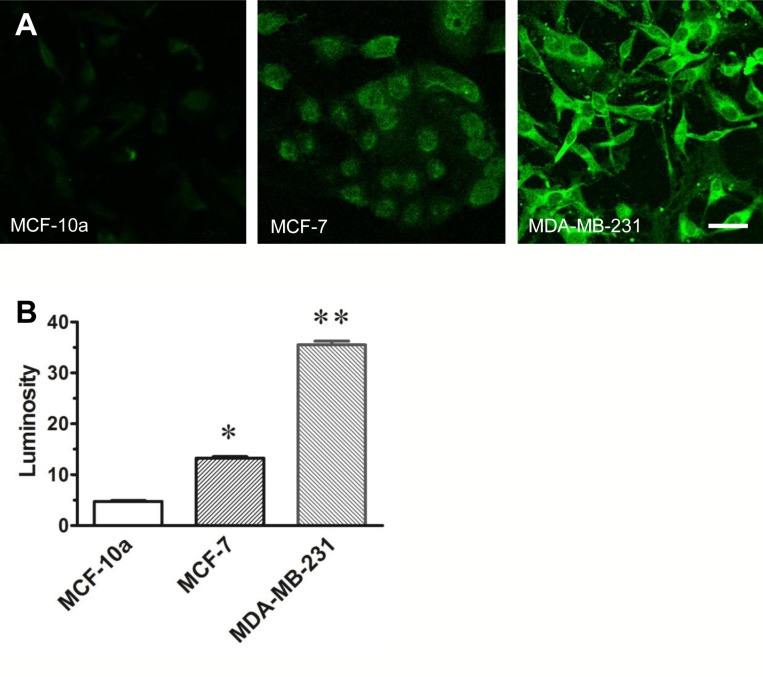
VGSC expression in MCF-10a, MCF-7 and MDA-MB-231 cells The photomicrographs (**A**) in A depict immunocytochemical labeling of VGSC protein observed in normal breast cells (MCF-10a), weakly-malignant (MCF-7) and highly-malignant (MDA-MB-231) breast cancer cells. The bar graph, (**B**) depicts the relative pixel counts obtained from 120 cell profiles outlined in confocal images of normal breast cells, weakly-malignant and highly-malignant breast cancer cells grown in culture that were processed with a pan-specific antibody that recognizes a conserved segment of the channel protein. Note that the results of the pixel evaluation of isolated cell profiles assessed in each cell line are consistent with relative field luminosity data depicted in A and the amounts of sodium channel protein directly correlates with the level of malignancy. *P* values compared to normal MCF-10a; ^*^p < 0.05; ^**^p < 0.01.

The relative immunofluorescent labeling of VGSC protein observed in normal and malignant breast cells is presented in Figure 1A. The relative intensity of the labeling seen in both the weakly-malignant (MCF-7) and highly-malignant (MDA-MB-231) breast cancer cells is 3–7 times greater respectively than that observed in normal breast cells (MCF-10a). The relative density of channel labeling as measured by pixel luminosity measurements is directly proportional with the level of malignancy (Figure 1B).

### Targeted osmotic lysis of cancer cells *in vitro*

**Figure 2:**
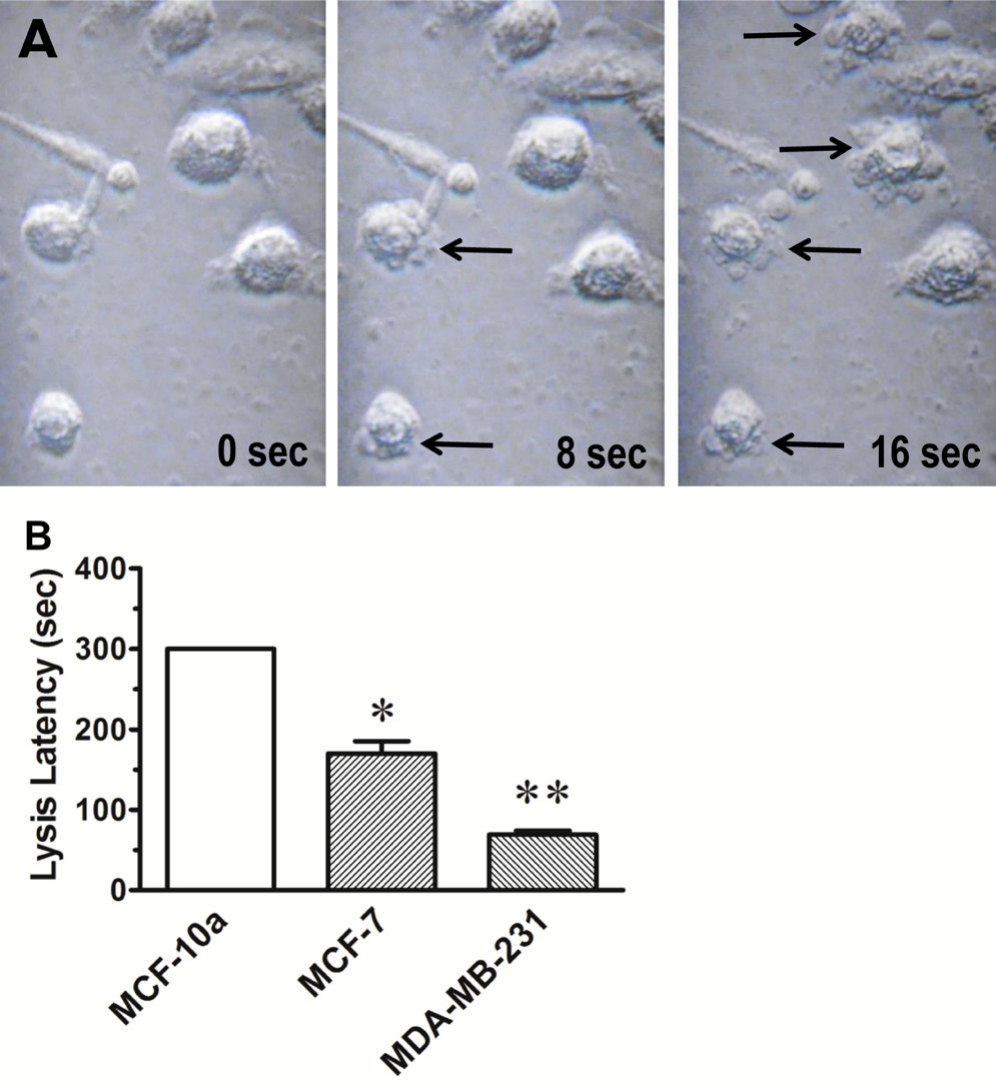
Osmotic lysis of cancerous breast cells (**A**) The photomicrographs were taken from best response video recordings that depict interval changes in cellular integrity of MDA-MB-231 cells in culture that were treated with ouabain (100 nM) and then stimulated with 1.0 V DC delivered in 1 msec pulse trains at 200 pps at stimulus onset (time 0) and after 8 sec and 16 sec of continuous stimulation. Note the bulging of cell membranes and the exudation of cytosol (arrows) into the surrounding media that is indicative of cell lysis. (**B**) The histogram depicts the average time to lysis of MCF-10a, MCF-7 and MDA-MB-231 cells treated with TOL. The results are consistent with the hypothesis that the rate of TOL is dependent on the relative number of sodium channels expressed in each of the cell types. Note that TOL spares the cells derived from normal breast tissue (MCF-10a). ^*^p < 0.05; ^**^p < 0.01.

The series of images depicted in Figure 2A, is illustrative of the process of cell lysis that occurs after applying continuous pulsed electrical stimulation to MDA-MB-231 cells in culture media containing 100 nM ouabain. The time to lysis of MDA-MB-231 cells was consistently less than 90 sec with an average time to lysis of 69.3 ± 4.2 sec (Figure 2B). All cells lysed within the 5 min stimulus time limit. By contrast, lysis of the MCF-7 cells required an average of 178 ± 12 sec of stimulation to lyse. Lysis of MCF-10a cells could not be achieved within the 5 min stimulus time limit. There was no evidence of change in cellular morphology in any of the cell lines produced by the TOL process other than the observed cell lysis.

### Sodium dependency of targeted osmotic lysis

When DMEM was replaced with normal Ringer’s solution, MBA-MB-231 cells (*n =* 24) lysed in a mean of 81.4 ± 8.3 sec. Lysis of MDA-MB-231 cells (*n =* 15) did not occur with stimulation times up to 5 min when DMEM was replaced with sodium-free Ringer’s solution (*p <* 0.001).

### Targeted osmotic lysis in breast, prostate, colon and lung carcinoma cell lines

Table 1 shows that carcinomas from tissues other than breast also lyse with this procedure. We assessed the time to lysis for neoplastic cell lines derived from prostate (LNCaP, DU145), colon (MCA-38), and lung (A549, 3LL) source organs, compared to replication for the two breast cancer lines. In addition, we compared the efficacy of digoxin to ouabain for each of these cell lines. Control treatments were vehicle-no stimulation, vehicle-stimulation, and drug-no stimulation.

**Table 1:** Time to lysis of cultured cancer cells

	VGSC expression^*^	Ouabain + Stimulation	Digoxin + Stimulation	No Drug, No Stim	Ouabain, No Stim	Digoxin, No Stim	Stim only
**Breast Cancer:**							
MCF-10a	+	300 (*n =* 10)	300 (*n =* 10)	300 (3)	300 (3)	300 (3)	300 (3)
MCF-7	++	170.5 ± 15.35 (*n =* 32)	180.3 ± 9.07 (*n =* 10)	300 (3)	300 (3)	300 (3)	300 (3)
MDA-MB-231	++++	69.3 ± 4.82 (*n =* 44)	68.39 ± 7.31 (*n =* 33)	300 (3)	300 (3)	300 (3)	300 (3)
Prostate Cancer:							
LNCaP	++	143.1 ± 5.78 (*n =* 28)	122.9 ± 3.13 (*n =* 21)	300 (3)	300 (3)	300 (3)	300 (3)
DU145	+++	81.6 ± 3.96 (*n =* 30)	111.3 ± 9.7 (*n =* 21)	300 (3)	300 (3)	300 (3)	300 (3)
Colon Cancer:							
MCA-38	++++	74.0 ± 1.0 (*n =* 2)	122.7 ± 9.02 (*n =* 3)	300 (3)	300 (3)	300 (3)	300 (3)
Lung Cancer:							
A549	++	149.9 ± 11.70 (*n =* 22)	179.8 ± 10.82 (*n =* 22)	300 (3)	300 (3)	300 (3)	300 (3)
3LL	+++	115.9 ± 3.33 (*n =* 31)	163.75 ± 8.27 (*n =* 24)	300 (3)	300 (3)	300 (3)	300 (3)

^*^Values from Onkal and Djamgoz, 2009; + = normal, ++ = ≥250-fold, +++ = ≥500-fold, ++++ = ≥1,000-fold over-expression of sodium channel RNA.

Seven carcinoma cell lines and one noncancerous cell line originating from four different tissue types were incubated in media alone or media + ouabain (100 nM) or media + digoxin (1 μM), and either stimulated or not stimulated. Time to lysis was determined by appearance of a visible split in the cell surface or evidence of cytosolic exudate extending from the base of the cell into the adjacent media. Mean time-to-lysis ± SEM was calculated for each group and are expressed in seconds. A value of 300 sec was used for all controls for statistical purposes, because none of the cells from control conditions lysed within the 5 min time limit. All drug + stimulation values for carcinoma lines were compared to no drug + no stimulation, *p <* .01.

As depicted in Table 1, 100 nM ouabain or 1 µM digoxin (the reported relative potency) combined with the pulsed electric current produced lysis in all of these carcinoma cell lines. Moreover, time to lysis was inversely correlated to the over-expression of sodium channels reported by Djamgoz and his colleagues. The Spearman correlation coefficients are r_s_ = –0.96, *p <* 0.001 for ouabain and –0.85, *p <* 0.01 for digoxin. Lysis was not observed in normal breast cells (MCF-10a) or any of the three control treatments.

### *In vivo* lysis of xenografted tumors

**Figure 3:**
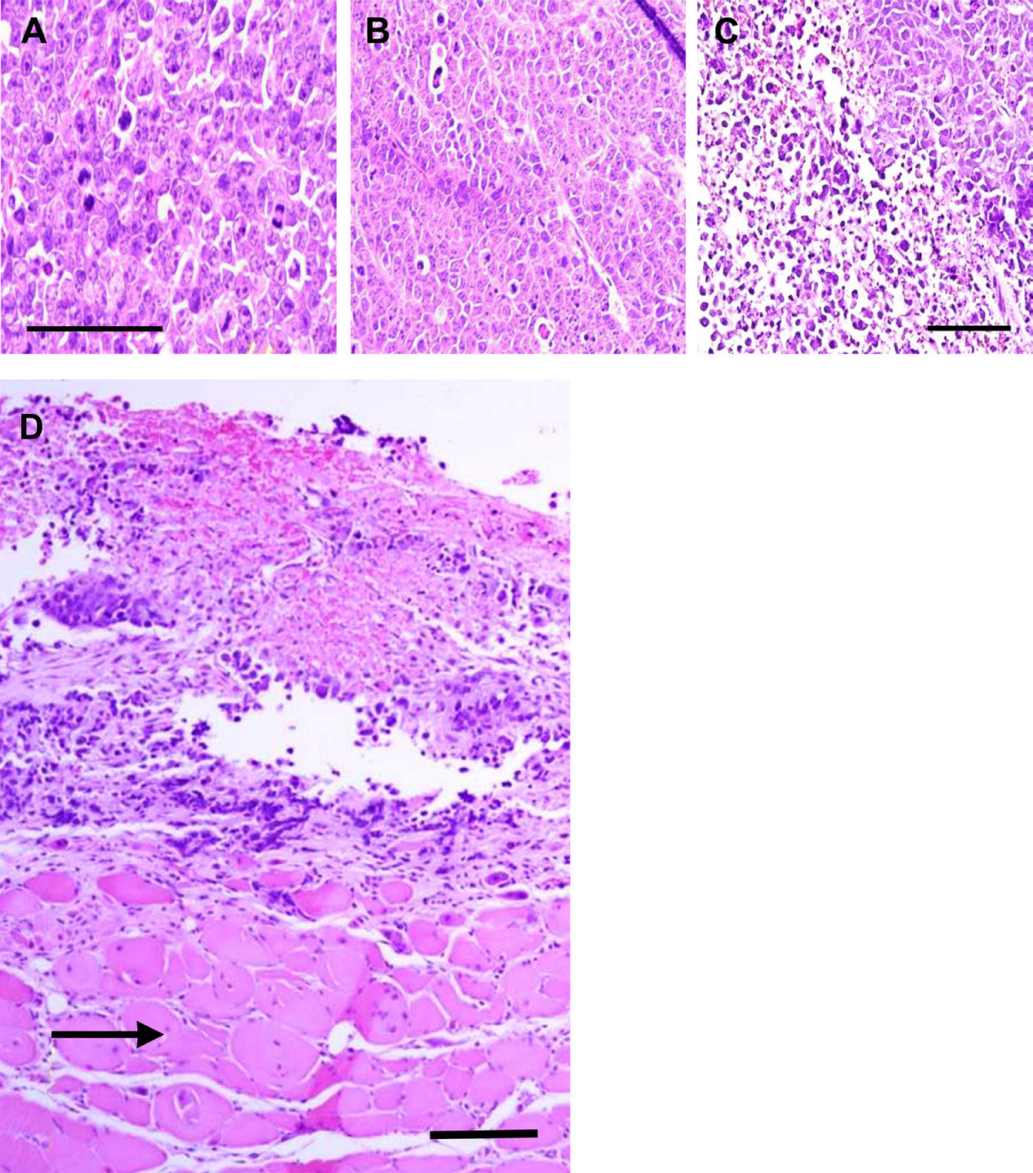
Histological verification of cell lysis after TOL Photomicrographs of malignant breast cancer xenografts that received no treatment (**A**), ouabain treatment alone (**B**) and ouabain followed by stimulation of 10 V, 200 pulses per second electric current twice for 1 min allowing 15 min without current between each stimulation (**C** and **D**). D includes uninjured muscle tissue (arrow) located adjacent to the tumor shown in C; calibration bars = 50 µm.

As depicted in Figure 3, tumors in each treatment group were stimulated electrically with 10 V, 200 Hz current twice for 1 min allowing 15 min without current between each stimulation. Destruction of neoplastic tissue in xenografted tumors of MDA-MB-231 cells ranged from 60–80% after TOL treatment (Figure 3C). Tumors from the ouabain-no stimulation (Figure 3B) and saline-stimulation groups (Figure 3A) showed no sign of lysis. Muscle cells adjacent to the lysed tumor cells showed no sign of lysis (Figure 3D).

### The effect of targeted osmotic lysis on tumor growth and survival

**Figure 4:**
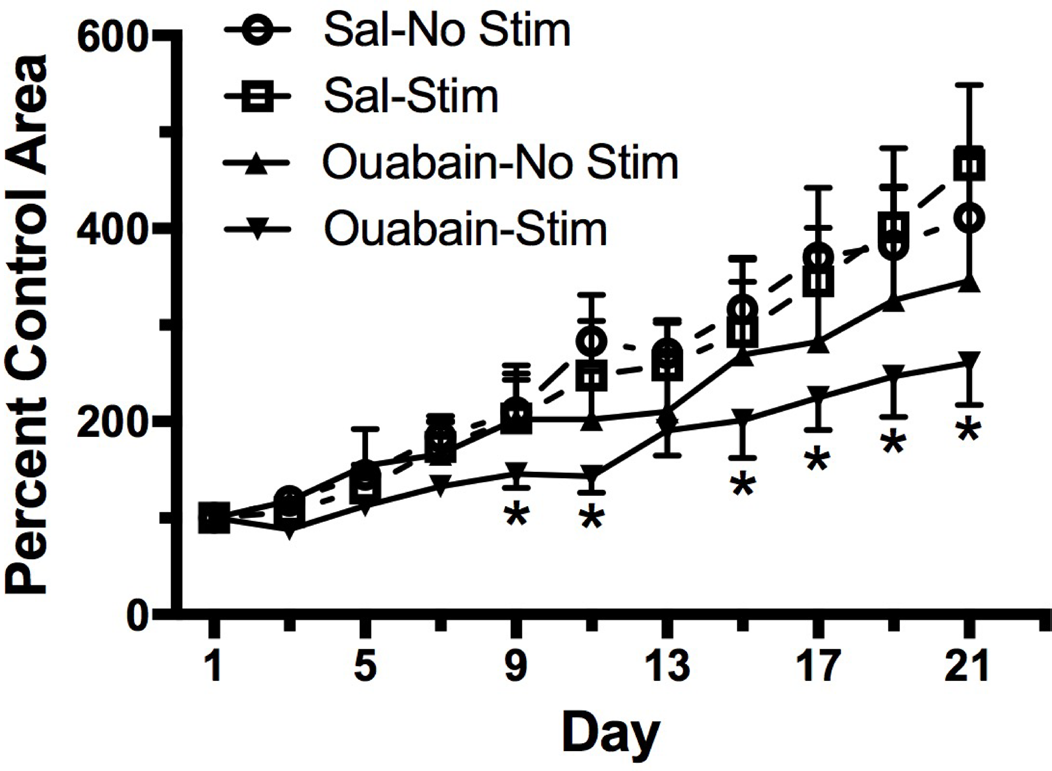
Survival and tumor growth after a single treatment with TOL Groups of male Nu/J mice (*n =* 6) were injected with MDA-MB-231 cells to establish xenografts. The graph illustrates the growth of xenografts in mice treated with TOL compared to the growth of xenografts in mice treated with saline (Sal), ouabain (10 mg/kg) or stimulation alone. All TOL-treated mice survived to the end of the experiment. One mouse in the saline group and 1 mouse in the Stim group met NIH criteria for euthanasia and were sacrificed early. ^*^p < .05 compared to controls.

**Figure 5:**
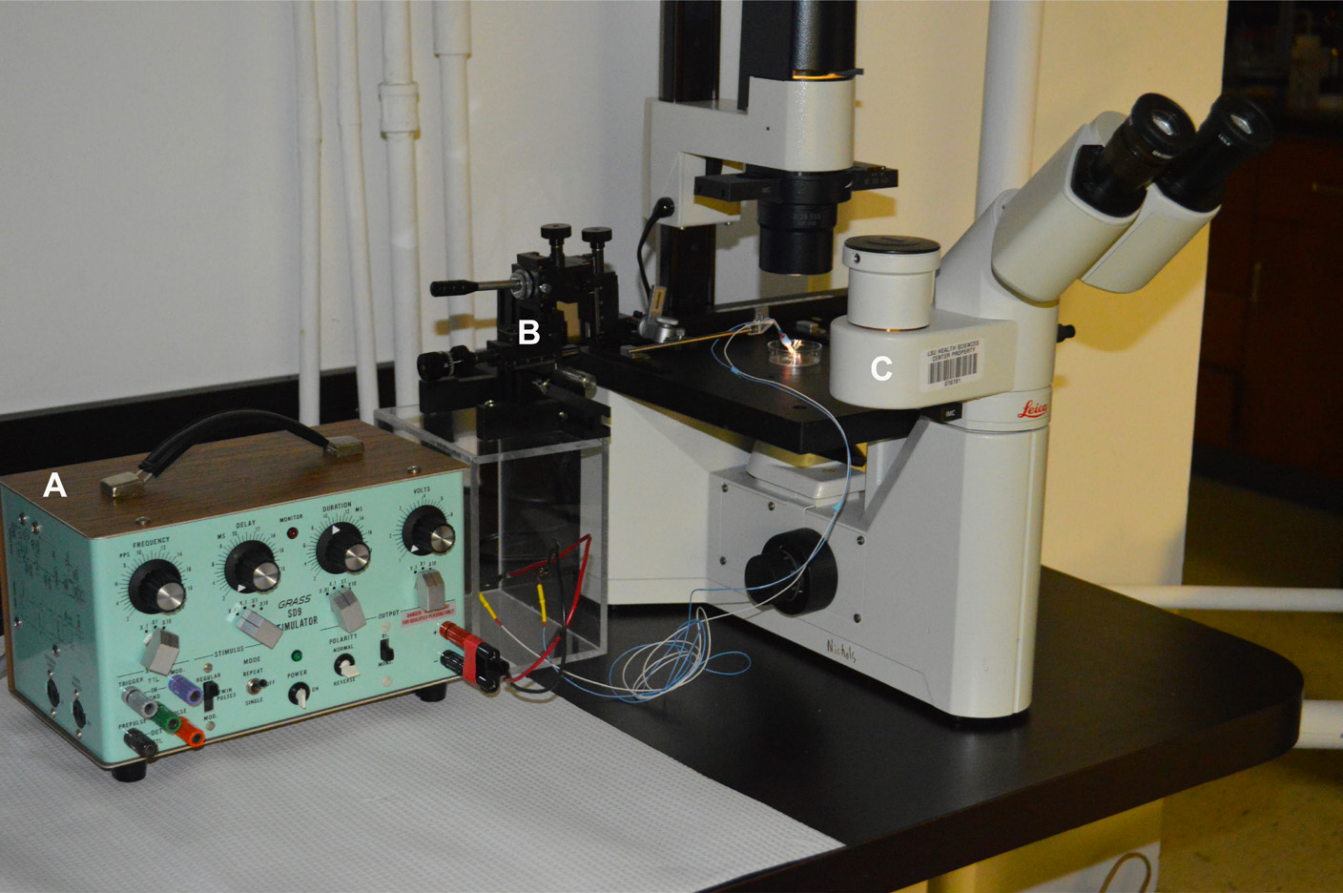
Photographic depiction of the apparatus used to stimulate cells *in vitro* (**A**) SD9 Grass Stimulator; (**B**) Narishige Model MN-151 Micromanipulator; (**C**) Leica Model DMIL Microscope.

To measure the effect of TOL on tumor mass and survival, groups of mice with MDA-MB-231 xenografts were treated with ouabain or normal saline vehicle as in the previous experiment. Tumor size was measured with digital calipers on the day of treatment and every other day thereafter. Figure 4 illustrates the growth of tumors in mice that received vehicle-no stimulation, ouabain-no stimulation, vehicle-stimulation, or a single treatment of ouabain-stimulation. Three weeks after treatment, the tumor mass in untreated mice had grown more than 3-fold. Tumors in mice treated with stimulation only were not significantly different from those in untreated mice. By contrast, the tumors in mice treated with ouabain only showed a small reduction in tumor mass when compared to untreated controls, but the tumors in the TOL-treated mice were substantially smaller than untreated controls at the end of 3 weeks. One mouse from the vehicle-treated group and one mouse from the group that received stimulation alone met the NCI criteria for euthanasia and were sacrificed prior to the end of the pre-determined survival period. None of the mice in the ouabain-no stimulation or ouabain-stimulation groups required early euthanasia. The stimulation set-up is depicted photographically in Figure 5.

## DISCUSSION

In this series of experiments, we have provided *in vitro* and *in vivo* evidence that the concomitant stimulation of VGSCs and Na^+^, K^+^-ATPase blockade will cause neoplastic breast, prostate, colon and lung cells that over-express VGSCs to lyse within seconds to minutes, whereas cells that express VGSCs at normal levels are relatively unaffected by such treatment. Eliminating sodium from the buffer blocked lysis, whereas in normal Ringer’s solution, cells lysed in times comparable to lysis times seen for these cells in DMEM. This is evidence that sodium is critical in producing the effect. The expression of channel protein was not as great as that reported for the over-expression of mRNA [4–6], but, as predicted, the difference in channel protein labeling observed is consistent with the relative level of malignancy.

Histologically, two 5 min TOL treatments produced lysis in 60–80% of cells in each treated tumor. With this treatment, tumor growth was reduced by more than 60% over a three-week post-treatment period.

When electric stimulation increases the opening of VGSCs and the conduction of sodium ions and extrusion of these ions by Na^+^, K^+^-ATPase is blocked by the cardiac glycoside drugs, the intracellular concentration of sodium approaches that of the extracellular fluid compartment, causing an increase in osmotic pressure. Water then passively follows the sodium across the cell membrane by osmosis, or through aquaporins, causing cellular swelling. Because the rigidity of cellular membranes is approximately that of a soap bubble, the cells that over-express VGSCs lyse. Cells with normal VGSC expression conduct less sodium, and thus less of an increase in intracellular osmotic pressure occurs. Therefore, less water enters the normal cells and they survive the treatment. Evidence for this mechanism is that a compromise in membrane integrity was observed and cytosolic exudate was present in the adjacent media within seconds to minutes after beginning treatment (Figure 2). Exclusion of Na^+^ from the test media prevented lysis and established sodium as a key factor in TOL.

TOL is a fundamentally different approach to the treatment of cancer than current treatments, both in principle and design. It takes advantage of a normal and conserved biological process that is essential for the maintenance of the proper intracellular sodium concentration for cell function and homeostasis. The process of maintaining proper intracellular/extracellular sodium balance is important for all cells but is exquisitely developed in excitable cells (e.g., nerve and muscle) for the preservation of their specialized function and for the maintenance of cellular integrity and adjusting to changes following injury and during wound healing [8–11]. Curiously, VGSCs are over-expressed in many epithelially-derived carcinomas [5, 6]; a feature that increases their ability to invade normal tissue and to spread throughout the body [12–17]. The application of traditional methods to destroy or compromise the function of the over-expressed sodium channels has been successful in decreasing tumor spread [5, 6, 18–21]. Unlike immunotherapies, TOL does not attack or destroy novel tissue markers or cells with these markers, but utilizes and facilitates activity in a portion of an over-expressed system to affect a treatment. The over-expression of VGSCs in breast and many other malignant carcinomas [5, 6, 18] identifies these cancers as potential targets for TOL. By enhancing sodium conductance while blocking its primary opposing restorative mechanism, TOL has the potential to achieve extraordinarily high efficacy and selectivity without significant morbidity. The conserved nature of these sodium handling mechanisms increases the potential for broad application of TOL.

Others have demonstrated that cardiac glycoside drugs induce apoptosis [22–24]. TOL differs from these studies in several ways. First, apoptosis is induced only after hours or days of exposure to the Na^+^, K^+^-ATPase blocking treatment, whereas TOL requires only enough time for the drug to bind to the pumps. Second, normal breast cell lines (MCF-10a) are more sensitive to cardiac glycoside-induced apoptosis than are some highly invasive lines, such as MDA-MB-231 [22], whereas the opposite is true of TOL. Third, the mechanism for cardiac glycosides to induce apoptosis involves activation of the Rho kinase [23, 24] and/or SRC pathways [22], whereas TOL is induced by excess intracellular Na^+^ concentration and the induction of osmotic lysis. The positive results observed in our *in vitro* studies, coupled with complementary results of the *in vivo* experiments provide evidence warranting further study of targeted osmotic lysis in experimental animals as a potential treatment for advanced stage carcinoma.

In summary, TOL may prove to be an effective and safe treatment for most forms of aggressive and invasive epithelially-derived carcinomas. Because over-expression of VGSCs is greatest in highly-malignant tumors, TOL is likely to be most efficacious in advanced and end-stage disease. When treatments of advanced carcinomas have failed or have been found to be largely ineffective, targeted osmotic lysis, an entirely new approach to treatment, may prove to be effective in fighting the late stages of many of the most deadly cancers.

## MATERIALS AND METHODS

### Drugs

Ouabain, digoxin, cholera toxin, and bovine insulin were purchased from Sigma (www.sigmaaldrich.com; #O3125, #D6003, #C8052, #I6634, respectively) and dissolved in culture media. CaCl_2_, KCl, N-[2-hydroxyethyl] piperazine-N;-[2-ethanesulfonic acid] (HEPES), sucrose and paraformaldehyde were also purchased from Sigma (#C5670, #P5405, #H3375, #S9378, #P6148, respectively). NaCl, MgSO_4_, and D-glucose were purchased from EMD (www.emdmillipore.com; #SX0420-3, #106067, #346351, respectively).

### Cell culture

MDA-MB-231 (ATCC, #HTB-26), MCF-7 (ATCC, #HTB-22), MCF-10a (ATCC, #CRL-10317), LNCaP (ATCC, #CRL-13009), DU145 (ATCC, #HTB-81) and A549 (ATCC, #CRM-CCL-185) cells were purchased from ATCC (www.atcc.org). The Stanley S. Scott Cancer Center Cell Culture Core Laboratory generously provided MCA-38 and 3LL cells. MDA-MB-231, MCF-7, and DU145 cells were cultured in T75 flasks containing high glucose Dulbecco’s modified eagle’s medium (DMEM; Gibco, Thermo Fischer Scientific, www.thermofisher.com; #11995–065) supplemented with sodium pyruvate, glutamine, 10% fetal bovine serum (FBS; Gibco: #16000044), and penicillamine/streptomycin (pen/strep; Gibco: #15140122). LNCaP, and 3LL cells were cultured in RPMI 1640 media (Gibco; #11875-093) supplemented with FBS and pen/strep. A549 cells were cultured in DMEM/F-12 media (Gibco; #11320-033 ) supplemented with FBS and pen/strep. MCF-10a cells were cultured in modified epithelial growth media (MEGM; Lonza/Clonetics, www.lonza.com) supplemented with 100 ng/ml cholera toxin, 10% fetal bovine serum and pen/strep.

Normal Ringer’s solution was prepared by dissolving in water the following substances at the specified concentrations: 125 mM NaCl, 5.0 mM KCl, 2.0 mM CaCl_2_, 1.0 mM MgSO_4_, 10.0 mM glucose, 10.0 mM HEPES. For sodium-free Ringer’s solution, the NaCl was replaced with 250 mM sucrose to maintain osmotic pressure.

### Animals

All animal experiments were approved by the LSU Health Sciences Center – New Orleans Institutional Animal Care and Use Committee and were in accordance with the NIH/NCI guidelines for use of animals in cancer research. Male Nu/J immuno-compromised mice (25–35 g) were purchased from Jackson Labs (www.jax.org; #002019) and housed in groups of 5 with *ad libitum* access to mouse chow and water. The mice were maintained in a temperature and humidity controlled colony with a 12/12 hr light/dark cycle. Xenografts of MDA-MB-231 cells were established by s.c. injection of 4–5 million cells suspended in 50% DMEM/50% matrigel (Corning, Inc., www.corning.com: #356237) into the dorsal trunk region between the scapulae.

### Over-expression of VGSCs

The relative cell surface expression of VGSCs was determined in normal breast cells (MCF-10a) and in weakly-malignant (MCF-7) and highly-malignant (MDA-MB-231) breast cancer cells grown from primary cell lines using immunohistofluorescence technology [9]. For imaging, cells were dissociated from the flasks with 0.25% trypsin-EDTA (Gibco, #25200056) and transferred to eight-chambered microscope slides (Nunc Lab-Tek II; Thermo Fischer Scientific, www.thermofisher.com) containing DMEM with 10 µM insulin. Insulin was added to insure normal sodium pump expression and function [25]. 24–48 hrs later, the DMEM was removed and the cells were fixed for 10–15 sec with 4% paraformaldehyde in 0.1 mol/L phosphate buffer, pH 7.6. After removal of the paraformaldehyde, the cells were washed with phosphate buffered saline and then immersed and incubated overnight at room temperature with a 1:100 dilution of a pan-specific VGSC antibody, EO3, (graciously provided by S. Rock Levinson, University of Colorado School of Medicine, Denver, CO). The EO3 antibody recognizes an epitope of the α-subunit that is conserved in all isoforms of VGSCs [26]. After removal of the primary antibody, the cells were rinsed with 0.1 mol/L phosphate buffered saline and then were incubated for 1 hour at room temperature with a 1:200 dilution of a goat-anti-rabbit secondary antibody conjugated to the Alexa Fluoro-488 fluorophore (Invitrogen, www.thermofisher.com: #A-11008). Cells incubated with the secondary antibody alone served as controls for nonspecific labeling. Cells were imaged using a HC PL APO CS2 20X/0.75 DRY objective lens on a Leica SP-2 confocal microscope (www.leica-microsystems.com). All images were produced using identical exposure times and imaging parameters. Microscopic analysis was performed in the xyz mode, with a 1.27 zoom and a 56.6µm pinhole (pinhole Airy = 1.00 Au). Excitation was initiated using a 488 nm (18.2%) line of an argon laser, On at 0.0000%, and photomultiplier tube set to detect an ALEXA 488 band-width, with a gain of 1241.7 and an offset of 0.13. To quantify immunohistofluorescence labeling, cellular profiles were outlined, and measurements of the mean number of pixels in each profile (luminosity) were made by using Adobe Photoshop CS4 (Adobe Systems, www.adobe.com). Statistical analysis of luminosity data was performed by using ANOVA with p set at 0.05.

### *In vitro* lysis of breast cancer cells

MCF-10a, MCF-7, and MDA-MB-231 cells were dissociated with trypsin and centrifuged for 5 min at 300Xg. The cells were re-suspended in the appropriate media, transferred to eight chambered microscope slides and then were returned to the incubator for 24–48 hrs before testing. Cells were grown to non-confluence prior to each experiment. DMEM was supplemented with 10 µM insulin. Ten to 15 min before testing, the media were replaced with media containing 100 nM ouabain and 10 µM insulin. With microscopic guidance (Leica model DMIL), electrodes were placed 1 mm apart near or on opposite sides of groups of cells using a Narishige model MN-151 micromanipulator (www.narishige-group.com). 1.0 V DC was delivered in continuous 1 msec pulse trains at 200 pps for up to 5 min using a Grass SD9 stimulator (Grass Instrument Company, Warwick, RI). The stimulation set-up is depicted photographically in Figure 5. Experiments were digitally recorded using a video camera attached to the microscope. Time to lysis of cells between the electrodes was recorded and mean time to lysis (± SEM) was calculated. Three controls were conducted: no treatment; ouabain treatment with no stimulation, and stimulation with no ouabain treatment. The media for all three controls also contained 10 µM insulin.

To confirm that the influx of sodium was responsible for the lysis of cancer cells in the TOL process MDA-MB-231 cells were cultured in 6-well plates. DMEM was replaced with Ringer’s solution with or without sodium 4 hrs before testing. Ouabain was then added to the media to a final concentration of 100 nM and the cells were stimulated and monitored as described above.

### *In vivo* lysis of xenografted tumors

The xenografted tumors were found variably and widely distributed under the skin over the dorsal surface of the trunk from the caudal neck to the sacrum. When the tumors reached 0.75–1.0 cm and appeared well vascularized (appeared red-violet through the skin), the mice were treated with saline or 10 mg/kg ouabain injected s.c. Thirty min later, the mice were anesthetized with isoflurane, an incision was made over the tumor, and a 10 V, 200 Hz DC current was applied continuously across the length of the tumor for 1 min. This procedure was repeated 15 min later and then the incision was closed with 5–0 silk sutures. One day later, the mice were sacrificed, perfused with buffered neutral formalin (BNF) and the tumors excised and stored in BNF until processing for hematoxylin and eosin staining. A pathologist blinded to the treatment protocol determined the percent of tumor lysis.

### Tumor growth and survival

To test the effect of TOL on tumor growth, mice with vascularized tumors were assigned to one of four treatment groups: saline-no stimulation; saline-stimulation; ouabain-no stimulation; and ouabain-stimulation. Mice in the ouabain-no stimulation and ouabain-stimulation groups were injected s.c. between the scapulae with 10 mg/kg ouabain. The saline-no stimulation and the saline-stimulation groups received an equal volume of normal saline by s.c. injection. Thirty minutes later, all mice were anesthetized with isoflurane. The tumors were measured with digital calipers at their point of greatest length and width. Mice in the groups that were not to receive stimulation were returned to their cages. DC current (10 V, 1 msec pulses, 15 pps) was then administered to the stimulation groups transcutaneously along the tumor’s axis of greatest diameter for 2.5 min and then were stimulated for another 2.5 min perpendicular to the axis of the initial stimulation. The stimulus parameters used for the growth and survival study differed from those used in the acute *in vivo* lysis experiments that were performed to determine the effectiveness of TOL in animals. For these initial experiments the skin was opened and the tumors were stimulated directly by inserting electrode tips into the tumors. By contrast, xenografted tumors in the growth and survival study were stimulated transcutaneously to minimize potentially confounding effects associated with surgical anesthesia, infection, pain and wound healing. Transcutaneous stimulation, however, imposes a number of variables not encountered when the tumors are surgically exposed, including how to hold an unanchored tumor between electrode contact points and overcoming skin impedance that had to be worked out. The stimulus parameters used for the growth and survival experiments were selected because they produced the most reproducible results in a series of pilot studies that tested many electrode designs and stimulation parameters. The animals were returned to their cages for 30 min and then were re-anesthetized and stimulated as before. Mice in the groups that did not receive stimulation were returned to their cages and monitored until recovery. Tumors were measured with digital calipers every other day for a maximum of 3 weeks and tumor cross-sectional area determined as a measure of tumor growth. Mice that met NIH criteria for euthanasia were sacrificed early for ethical reasons.

### Statistics

Immunocytofluorescence experiments were analyzed using a one-way analysis of variance (ANOVA). Lysis experiments were analyzed using a mixed design ANOVA with Tukey post-hoc comparisons, or student’s *t*-test (sodium-free Ringer’s experiment). The tumor measurements were analyzed using a repeated measures, mixed design ANOVA to determine statistical significance between tumor growth in the TOL-treated tumors and those in the control groups. ANOVA with *p* set at 0.05.
